# A case of paroxysmal complete atrioventricular block in a COVID‐19 patient

**DOI:** 10.1002/ccr3.4268

**Published:** 2021-10-23

**Authors:** Hong Nyun Kim, Myung Hwan Bae, Bo Eun Park, Jaehee Lee

**Affiliations:** ^1^ Division of Cardiology Department of Internal Medicine Kyungpook National University Hospital Daegu Korea; ^2^ Department of Internal Medicine School of Medicine Kyungpook National University Daegu Korea; ^3^ Division of Pulmonology Department of Internal Medicine Kyungpook National University Hospital School of Medicine Kyungpook National University Daegu Korea

**Keywords:** acute respiratory distress syndrome, arrhythmia, COVID‐19, paroxysmal complete atrioventricular block, SARS‐CoV‐2

## Abstract

Many types of cardiac arrhythmias can occur in people with COVID‐19, and these arrhythmias can affect the patient's outcomes. We have experienced paroxysmal complete atrioventricular block in a patient with COVID‐19 and would like to share the course of treatment.

## Graphical abstract

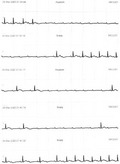

## 1 INTRODUCTION

A patient with coronavirus disease 2019 showed complete atrioventricular block on electrocardiogram. The patient was undergoing mechanical ventilator treatment for severe hypoxia. Intrathoracic pressure was reduced by adjusting the tidal volume and the positive end‐expiratory pressure of the mechanical ventilator. After that, complete atrioventricular block did not occur during the hospitalization.

Humanity is still at war with the coronavirus disease 2019 (COVID‐19) since the first outbreak of the severe respiratory syndrome coronavirus 2 in December 2019. Cases are still rising, and mortality is still occurring. Many clinical reports on COVID‐19 and various data about cardiac arrhythmia are in existence. However, the association with cardiac arrhythmia and its mechanism should be acknowledged. The COVID‐19 pandemic was experienced in Daegu, Korea, between March and May 2020. Several clinical features were experienced during the treatment of the COVID‐19 infection including cardiac arrhythmia. The first case of a paroxysmal complete atrioventricular block is herein presented to share the course of treatment.

## 2 CASE DESCRIPTION

**FIGURE 1:**
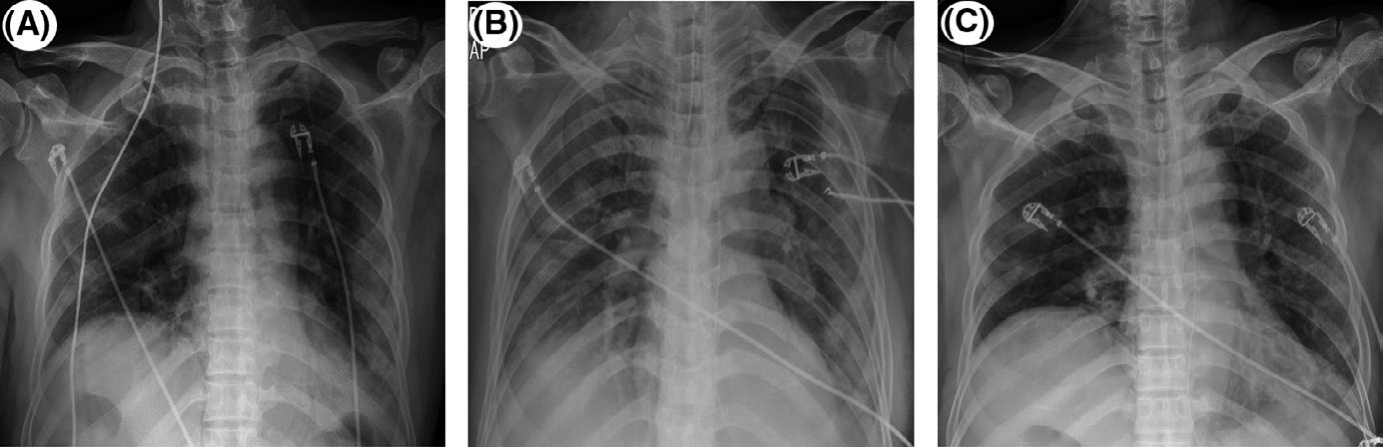
Chest X‐ray on the day of admission (A), day 6 (B), and day 13 (C) of hospitalization. Ground‐glass opacities and nodular opacities in the left middle and both lower lobes deteriorated on day 6 of admission and improved on day 13 of admission

**FIGURE 2:**
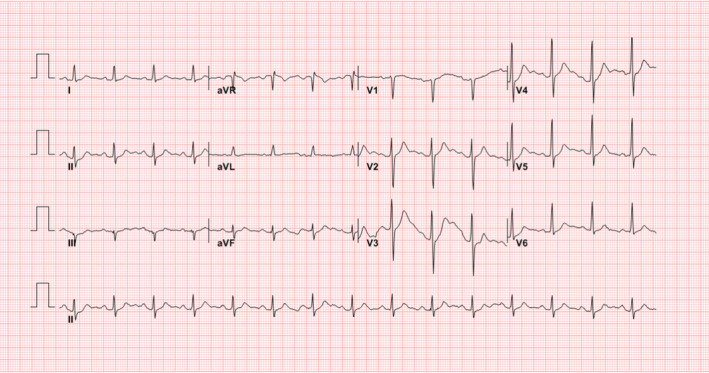
ECG on the day of admission. Initial ECG was normal for the QRS complex and PR interval

**FIGURE 3:**
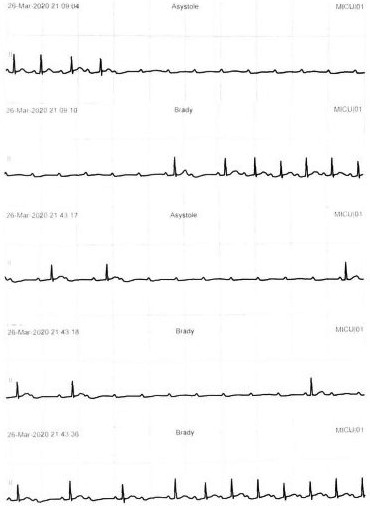
ECG of paroxysmal complete atrioventricular block. ECG strips presented paroxysmal complete atrioventricular block with slow escape ventricular beats

A 72‐year‐old male was transferred from a local hospital on 18 March 2020, due to severe hypoxia and hypercapnia. The patient was taking medication for hypertension and dyslipidemia and was a nonsmoker. He developed upper respiratory symptoms such as headache, fever, chilling sensation, and cough 8 days before being transferred to our hospital. A real‐time reverse transcriptase‐polymerase chain reaction test for COVID‐19 was done which confirmed COVID‐19 infection 5 days prior to being transferred to our hospital. He was an inpatient at a local medical institution and was treated with antibiotics (levofloxacin) and antiviral agent (lopinavir/ritonavir) 2 days before being transferred to our hospital. However, the patient's chest X‐ray haziness was aggravated, fever was sustained, and hypoxia worsened despite the symptomatic treatment. His initial vital signs were blood pressure of 136/63 mm Hg, heart rate of 79 beats/min, body temperature of 37.3℃, respiratory rate of 40 breaths/min, and peripheral O2 saturation of 90% on non‐rebreather mask of 15 L/min. The pH, pO2, pCO2, and O2 saturation were 7.318, 78.1 mm Hg, 50.7 mm Hg, and 93.8%, respectively, in the arterial blood gas analysis. Chest X‐ray demonstrated diffuse ground‐glass opacities in the left middle and both lower lobes (Figure 1). Initial electrocardiogram (ECG) revealed normal sinus rhythm with a heart rate of 90 beats/min, normal PR (172 ms) and QRS (92 ms) intervals, and normal QT (350 ms) and QTc (428 ms) intervals (Figure 2). The patient underwent intubation and was started on mechanical ventilator care 1 hour after admission. Medical treatment was administered with antibiotics (levofloxacin) and antiviral agent (darunavir/cobicistat). A mechanical ventilator was applied, and midazolam and fentanyl citrate were used for sedation initially. The heart rate was stably maintained at 60‐70 beats/min, and cardiac arrhythmia was not observed. On day 2 of hospitalization, antibiotics was changed from levofloxacin to piperacillin/tazobactam due to persistent fever. On day 4 of admission, the antiviral agent was stopped due to liver enzyme elevation. In addition, antibiotics (azithromycin) was added on day 6 of hospitalization because chest radiography demonstrated deteriorating pulmonary consolidation (Figure 1). On day 9 of admission, a paroxysmal complete atrioventricular (AV) block was observed on the ECG monitor (Figure 3). Paroxysmal complete AV block lasted for 5‐10 seconds at the time of occurrence and developed one to two times per hour. Changes in consciousness, such as syncope, were not observed because the patient was in the sedation state at the time of paroxysmal complete AV block onset. Accordingly, we first identified and discontinued medication that could induce cardiac arrhythmia. The antibiotics piperacillin/tazobactam and azithromycin were discontinued and were changed to a different class of antibiotics. In addition, the mechanical ventilator setting value was adjusted in consideration of the possibility of AV block due to vagal overstimulation due to an increase in intrathoracic pressure and hyperinflation of the lungs. At the time, the patient's mechanical ventilator was set to assisted/controlled mandatory ventilation—volume controlled ventilation mode with 340 mL per inspiration of tidal volume (TV), 10 cmH_2_O of positive end‐expiratory pressure (PEEP), 28 breaths/min of respiratory rate, and 50% fraction of inspiration oxygen.

**FIGURE 4:**
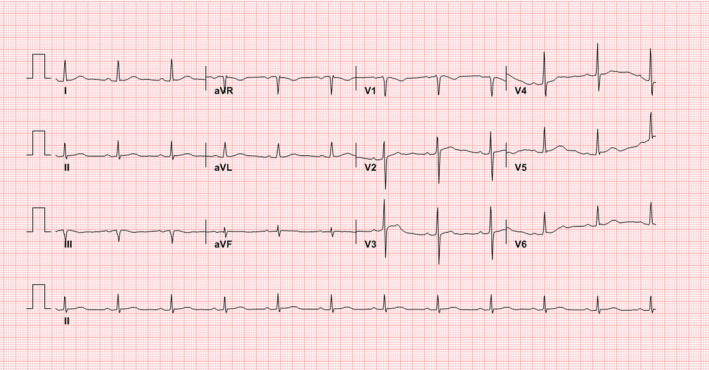
ECG after recovery from a paroxysmal complete atrioventricular block. The ECG showed normal sinus rhythm with a heart rate of 68 beats/min and normal PR and QRS intervals

TV and PEEP were adjusted from 340 to 320 mL per inspiration and from 10 to 8 cmH_2_O, respectively. Paroxysmal complete AV block did not occur 2 hours after adjusting the mechanical ventilator, and follow‐up ECG revealed normal sinus rhythm with a heart rate of 68 beats/min (Figure 4). Thereafter, cardiac arrhythmia including paroxysmal complete AV block was not observed during the hospitalization period. Fortunately, the patient gradually recovered from acute respiratory distress syndrome. On day 13 of hospitalization, mechanical ventilation was stopped and extubation was performed. Finally, the patient was discharged 49 days after admission.

## 3 DISCUSSION

Cardiac arrhythmia related to many viral infections including influenza virus,^1^ Zika virus,^2^ Epstein‐Barr virus,^3^ and human immune‐deficiency virus^4^ has been reported several times.^5^ A case also exists of a high‐degree AV block caused by the H1N1 influenza virus impacting the cardiac conduction system.^6^ Several types of tachyarrhythmia and bradyarrhythmia have been reported in severe acute respiratory syndrome and Middle East respiratory syndrome outbreaks that occurred before COVID‐19.^7^, ^8^ As a mechanism of cardiac arrhythmia, virus infection can induce myocardial injury, and damage to the conduction system can consequently trigger cardiac arrhythmias.^9^ In addition, systemic infection, hypoxemia, pre‐existing cardiac diseases, comorbidities, and advanced age affect the development of cardiac arrhythmia.

Various tachyarrhythmia and bradyarrhythmia have also been reported in COVID‐19 patients.^10^, ^11^ In a report from China, cardiac arrhythmia was observed in 16.7% and 44.4% of patients hospitalized for COVID‐19 and patients admitted to the intensive care unit, respectively.^12^ In COVID‐19 patients, high‐degree AV block such as complete AV block is rare. However, some cases have been reported.^13^, ^14^ It is speculated that cardiac arrhythmia may be caused by the aforementioned mechanisms and causes even in COVID‐19 patients. So, first, the conduction disturbances due to the injury of the myocardium after COVID‐19 infection can be considered. However, in the case of this patient, cardiac enzyme, troponin I (<0.015 ng/mL), was within the normal range at the time of the development of the paroxysmal complete AV block. In addition, it was difficult to clinically find evidence of myocardial injury. Therefore, the possibility of paroxysmal complete AV block due to damage to the conduction system is considered to be relatively low.

Second, the medication used in the patient possibly induces cardiac arrhythmia. In the patient of this study, piperacillin/tazobactam and azithromycin were used as antibiotics when inducing paroxysmal complete AV block. In addition, antiviral agent was not used due to elevated liver enzyme levels. Antimalarial drugs such as chloroquine and hydroxychloroquine have not been used in patients. It is known that piperacillin/tazobactam rarely causes hypokalemia and has the potential to develop torsade de pointes (TdP).^15^ However, evidence concerning the association with AV block is lacking. Azithromycin induces QRS widening and QRS prolongation, and it is known to induce serious ventricular arrhythmia such as TdP.^15^, ^16^ However, finding a correlation with the complete AV block was difficult. Sedatives used in patients are also likely to cause arrhythmia.^17^ When the patient developed CAVB, midazolam was continuously injected at 0.11mg/kg/hr Midazolam can induce ventricular ectopy, bradycardia, and prolongation of the QT interval.^18^, ^19^ However, it was difficult to find evidence that midazolam causes paroxysmal CAVB. In addition, there was no change in sedative before and after paroxysmal CAVB, and paroxysmal CAVB disappeared and did not recur in the course of the treatment despite continued use of midazolam.

Third, a mechanical ventilator was used in our patient for the treatment of severe hypoxia and hypercapnia when paroxysmal complete AV block developed. Mechanical ventilator treatment with higher plateau pressure and greater minute ventilation may be associated with agitation and delirium.^20^ Agitation and delirium have been associated with cardiac arrhythmia and increased mortality.^21^ In this patient, we applied deep sedation, Richmond Agitation‐Sedation Scale equal to – 4 points, because he had severe hypoxia. Therefore, no agitation was observed when the patient developed paroxysmal CAVB.

As aforementioned, the PEEP was applied to the patient and a high respiratory rate was maintained for correction of hypoxia and hypercapnia. The application of PEEP has the potential to increase intrathoracic pressure.^22^ In addition, the high respiratory rate applied simultaneously with PEEP induces dynamic hyperinflation of the lungs.^23^ Activation of the pulmonary C and J receptor occurs if the lungs are hyperinflated, which can lead to vagal stimulation.^24^ Moreover, excessive vagal stimulation causes a decrease in heart rate and blocks the conduction of the heart at the AV node. In consideration of this possibility, the PEEP and the tidal volume were reduced from 10 to 8 cmH_2_O and 340 to 320 mL per inspiration, respectively. Thereafter, paroxysmal complete AV block disappeared. No further occurrences were observed.

Finally, various types of arrhythmia have been reported in the treatment course of COVID‐19 patients. However, the relationship between COVID‐19 and arrhythmia still lacks objective evidence and an understanding of its mechanism. Paroxysmal complete AV block may also be associated with COVID‐19 infection but can be caused by the patient's conditions, comorbidities, and medications. Therefore, the aforementioned contents should be checked first if complete AV block occurs during the treatment of COVID‐19 patients. Moreover, amendments for correctable factors should be made in advance. Temporary cardiac pacing or permanent pacemaker implantation should be considered even after these measures if complete AV block persists or if the patient has severe hemodynamic impairment or severe bradycardia symptoms.

## CONFLICT OF INTEREST

All authors have no potential conflicts of interest to disclose.

## AUTHOR CONTRIBUTIONS

Bae MH: conceptualized the study. Kim HN: investigated the study. Kim HN: wrote original draft preparation. Bae MH, Kim HN, Park BE, and Lee JH: wrote review and editing. All authors approved the final manuscript.

## ETHICS STATEMENT

The study protocol was reviewed and approved by the Institutional Review Board of the School of Medicine, Kyungpook National University (IRB No. 2020‐07‐062), Daegu, Korea.
